# Supplementary material for: Microbial community analysis in the gills of abalones suggested possible dominance of epsilonproteobacterium in Haliotis gigantea
Source: PeerJ. 2020 Jun 30;8:e9326. doi: 10.7717/peerj.9326 (PMC7333650; doi:10.7717/peerj.9326)
Supplement: Supplemental Information 1 — Observed OTU number and Chao 1, Shannon, Simpson and Good’s coverage indices. [file peerj-08-9326-s001.docx]

| **Sample**  **ID** | **Shannon**  **Index** | **Observed**  **OTUs** | **Chao 1**  **index** | **Good's**  **coverage** |
| --- | --- | --- | --- | --- |
| Hgig1 | 3.80 | 120 | 120.00 | 100.0% |
| Hgig2 | 2.58 | 51 | 51.00 | 100.0% |
| Hgig3 | 3.37 | 77 | 77.00 | 100.0% |
| Hgig4 | 4.23 | 81 | 81.00 | 100.0% |
| Hgig5 | 3.17 | 89 | 89.00 | 100.0% |
| Hgig6 | 5.80 | 165 | 165.00 | 100.0% |
| Hgig avg. ± SE | 3.83 ± 0.42 | 97.17 ± 14.90 | 97.17 ± 14.90 | 100.0% ± 0 |
| Hdis1 | 5.40 | 183 | 183.00 | 100.0% |
| Hdis2 | 4.59 | 122 | 122.00 | 100.0% |
| Hdis3 | 5.80 | 155 | 155.00 | 100.0% |
| Hdis4 | 4.27 | 95 | 95.00 | 100.0% |
| Hdis avg. ± SE | 5.01 ± 0.31 | 138.75 ± 16.61 | 138.75 ± 16.61 | 100.0% ± 0 |
| Hdiv1 | 4.22 | 84 | 84.00 | 100.0% |
| SW | 4.49 | 117 | 117.00 | 100.0% |
| ST | 7.09 | 327 | 327.25 | 99.9% |
