# Supplementary figures and images for: Microbial community analysis in the gills of abalones suggested possible dominance of epsilonproteobacterium in Haliotis gigantea
Source: PeerJ. 2020 Jun 30;8:e9326. doi: 10.7717/peerj.9326 (PMC7333650; doi:10.7717/peerj.9326)

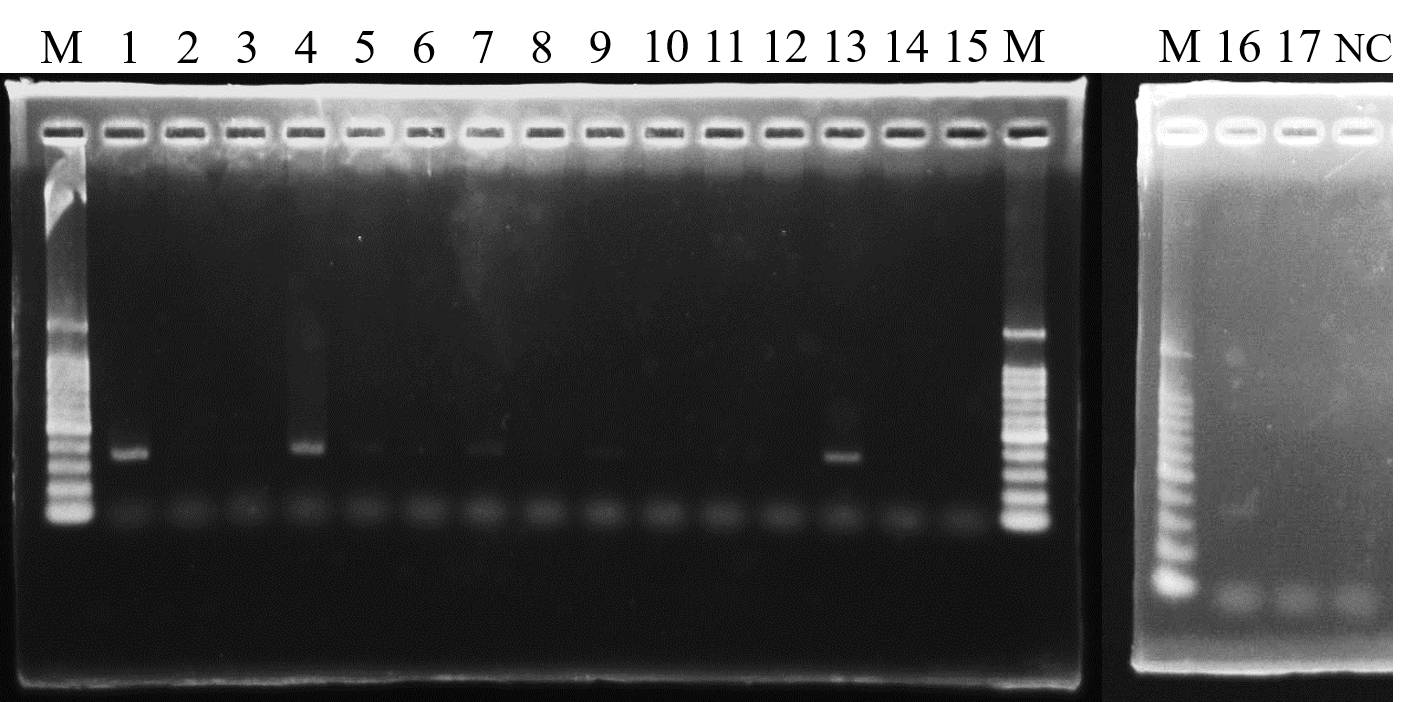

Supplement: Supplemental Information 2 — Lanes M, 100-bp DNA Ladder; lane 1: gill of Hgig2; lane 2: gut of Hgig2; lane 3: foot of Hgig2; lane 4: gill of Hgig3; lane 5: gut of Hgig3; lane 6: foot of Hgig3; lane 7: gill of Hgig4; lane 8: gut of Hgig4; lane 9: foot of Hgig4; lane 10: gill of Hgig5; lane 11: gut of Hgig5; lane 12: foot of Hgig5; lane 13: gill of Hgig6; lane 14: gut of Hgig6; lane 15: foot of Hgig6; lane 16: gill of Hgig1; lane 17: gut of Hgig1; lane NC: negative control. [file peerj-08-9326-s002.png]
